# BASP1 is a prognostic biomarker associated with immunotherapeutic response in head and neck squamous cell carcinoma

**DOI:** 10.3389/fonc.2023.1021262

**Published:** 2023-01-27

**Authors:** Xue Pan, Xun Xu, Lixuan Wang, Siyuan Zhang, Yingyao Chen, Rongchun Yang, Xijuan Chen, Bin Cheng, Juan Xia, Xianyue Ren

**Affiliations:** ^1^ Hospital of Stomatology, Sun Yat-sen University, Guangzhou, China; ^2^ Guangdong Provincial Key Laboratory of Stomatology, Guangzhou, China; ^3^ Guanghua School of Stomatology, Sun Yat-sen University, Guangzhou, China

**Keywords:** HNSCC, BASP1, immunotherapy, CD8^+^ T cells, ferroptosis

## Abstract

**Backgrounds:**

Immunotherapy is effective in a subset of head and neck squamous cell carcinoma (HNSCC). However, the unfavorable response rate and inadequate biomarkers for stratifying patients have primarily limited its clinical application. Considering transcriptional factors (TFs) play essential roles in regulating immune activity during HNSCC progression, we comprehensively analyzed the expression alterations of TFs and their prognostic values.

**Methods:**

Gene expression datasets and clinical information of HNSCC were obtained from The Cancer Genome Atlas (TCGA) and Gene Expression Omnibus (GEO) repository. Then, Brain abundant membrane attached signal protein 1 (BASP1) was screened out of differentially expressed TFs by univariate and multivariate survival analysis. Tumor immune dysfunction and exclusion (TIDE) was applied to analyze the response to immunotherapy of BASP1^high/low^ patients. Meanwhile, GO, KEGG and GSEA analyses were used to enrich the pathways between the BASP1^high^ and BASP1^low^ groups. Single-sample gene set enrichment analysis (ssGSEA), CIBERSORT, EPIC and quanTiseq algorithms were applied to explore immune infiltrations. Also, immune cycle analysis was conducted by ssGSEA. Additionally, lipid peroxidation, glutathione and reactive oxygen species were performed to detect the ferroptosis alternations.

**Results:**

BASP1 was upregulated and associated with poor survival in HNSCC patients. BASP1^high^ patients exhibited better response rates to anti-PD-1 immunotherapy and higher expressions of immune checkpoint inhibitors. GO, KEGG and GSEA analyses indicated that the expression of BASP1 was related to several immune-related pathways and immunogenic ferroptosis signature. The infiltration of activated CD8^+^ T cells was authenticated to be decreased in BASP1^high^ patients. Furthermore, BASP1 was identified to be positively correlated with T cell dysfunction and immune escape. Moreover, silencing BASP1 triggered ferroptosis in HNSCC cells, representing as increased LDH, lipid peroxidation and ROS levels, and reduced glutathione synthesis

**Conclusions:**

We demonstrated that BASP1 suppressed immunogenic ferroptosis to induce immunosuppressive tumor microenvironment. BASP1 plays a critical role in immune response, and might be a promising classifier for selecting HNSCC patients who benefit from current immunotherapy.

## Introduction

Head and neck squamous cell carcinoma (HNSCC) is the 7th most common malignant tumor worldwide with high mortality (1). Immune checkpoint inhibitors (ICIs) therapies that recover T cell cytotoxicity effect towards tumor cells have achieved remarkable progress in multiple cancers (2–4). The programmed cell death 1 (PD-1) antibodies, pembrolizumab and nivolumab, have been approved for the first-line treatment of recurrent/metastatic HNSCC patients since 2019. Although the responses seem to be durable to those who benefit from these agents, only a subset of HNSCC patients is expected to respond to ICIs for the lack of reliable predictive biomarkers (5, 6). Hence, the importance of developing reliable predictive biomarkers for immunotherapy and ideal therapeutic strategies for personalized clinical management of HNSCC patients has been highlighted (7).

HNSCC tumors exhibit high heterogeneity of tumor microenvironment (TME) and evade immune surveillance by a number of different mechanisms (8). The immune cells in the TME consist of tumor-infiltrating lymphocytes (TILs), including natural killer (NK) cells, T cells and B cells, and myeloid-lineage cells, including dendritic cells, neutrophils, and macrophages. The effector CD8^+^ T cells and NK cells are the main components of immune killing and tumor cell elimination, while Treg cells and M2 macrophages are responsible for immune suppression and tumor progression. Molecular signatures and biomarkers have been constructed as classifiers to identify immune phenotypes of HNSCC tumors for predicting ICIs therapy response, including epithelial-mesenchymal transition (EMT), ferroptosis, etc. (9, 10). However, HNSCC patients who might respond to ICIs therapy have not yet been identified.

Transcription factors (TFs) play a leading role in the initiation of cancer progression *via* regulating the expressions of cancer hallmarks genes (11), such as DNA damage and repair, EMT, and immune response, and are potential prognostic biomarkers and therapeutic targets for developing anticancer drugs (12). Our study has demonstrated that SPDEF could transcriptionally activate NR4A1 to suppress the HNSCC progression (13). Additionally, a recent study has mentioned that TYRO3 is a predictive biomarker for patient stratification in breast cancers to suppress immune therapeutic outcomes by limiting tumoral ferroptosis (14). TCF7 has been recognized as a practical marker to predict positive clinical outcomes in patients treated with anti-PD-1 therapy (15). However, the prognostic prediction values and biological functions of TFs in HNSCC remain unclear.

In this study, we comprehensively explored the aberrantly expressed TFs in HNSCC and their correlation with the survival of HNSCC patients. We identified that BASP1 was upregulated with poor prognosis, and correlated with positive response to ICIs therapy. We then performed functional enrichment and immune cell infiltration analyses between the BASP1^high^ and BASP1^low^ patients. Our results indicated that BASP1 was associated with immune cell infiltration and ferroptosis in HNSCC and could predict prognosis and anti-PD-1 therapeutic response, which might offer a novel therapeutic strategy for HNSCC patients.

## Materials and methods

### Datasets and data preprocessing

RNA sequencing (RNA-seq, count and TPM values) and clinical data were downloaded from the HNSC dataset of The Cancer Genome Atlas (TCGA) database and calculated using TCGAbiolinks R package (16). Transcripts per kilobase million (TPM) values of RNA-seq data were used to compare the differences in gene expression between normal (n=44) and tumor (n=502) samples (17). Cases with insufficient or missing data were deleted from subsequent data processing. GSE30784, GSE103322, GSE41613 and GSE65858 were downloaded from the Gene Expression Omnibus (GEO) repository with GEOquery R package (18–22). GSE30784 and GSE103322 were used to analyze differential expression genes, while GSE41613 and GSE65858 were used to validate survival analyses. Furthermore, The TFs list was collected from AnimalTFDB 3.0 (http://bioinfo.life.hust.edu.cn/AnimalTFDB/) (23). Genes encoding immunomodulators and chemokines and gene signatures of TILs were obtained from TISIDB (http://cis.hku.hk/TISIDB). Ferroptosis scores referred to previous study (24).

### Differential expression analysis of TFs

To obtain genes differentially expressed between tumor and normal tissue, differentially expressed genes (DEGs, adjusted P-value < 0.05, |log_2_FC| > 1) were screened by DEseq2 package with the raw count data of HNSCC samples. The limma package was used to analyze the differentially expressed TFs (DE-TFs) in GSE30784 and GSE103322 with the threshold set at false discovery rate (FDR) < 0.05 & |log2FC| >1 and FDR < 0.05 & |log2FC| > 0.4, respectively. The intersection of three DE-TFs sets was achieved and the Venn plot was drawn with Venngram package.

### Prognosis analysis

Univariate Cox regression and the Kaplan-Meier method were used to assess the prognostic role of DE-TFs in the overall survival (OS) of HNSCC patients. The continuous variable of DE-TFs TPM data was used in the univariate Cox regression. Then, candidates with p < 0.05 were entered into stepwise multivariate Cox proportional hazard regression models. High and low groups based on BASP1 expression (BASP1^high^ and BASP1^low^) were applied to perform Kaplan–Meier curves analysis, employing survminer and survival packages. The best cut-off points were evaluated by the “surv-cutpoint” function in the survminer R package. The log-rank P-value and hazard ratio (HR) with 95% confidence intervals were calculated and the survival analysis outcomes were presented as forest plots, tables and Kaplan-Meier plots, respectively.

### Gene function analysis

DEGs (adjusted P-value < 0.05) between low- and high- BASP1 expression groups were identified by the DESeq2 package. Then, DEGs with a significant correlation with BASP1 (p < 0.05, |r| > 0.2) were included in the Gene Ontology (GO), Kyoto Encyclopedia of Genes and Genomes (KEGG) and Gene set enrichment analysis (GSEA) analyses using ClusterProfiler package (25). The adjusted p < 0.05 was set in GO and KEGG analysis, and bubble plot and bar plot were applied to show the outcomes. Furthermore, the “gmt” file of the hallmark gene set (h.all.v7.5.1.entrez.gmt) was obtained from Molecular Signatures Database (MSigDB, https://www.gsea-msigdb.org/gsea/index.jsp). FDR < 0.05, and normalized enrichment score (|NES|) > 1 were considered significantly different and shown by enrichplot package.

### Immune features analysis

The Tumor Immune Dysfunction and Exclusion (TIDE, http://tide.dfci.harvard.edu/) (26) algorithm was employed to evaluate the exclusion of CTLs. And the evaluation of BASP1 as a biomarker was obtained from the TIDE website. Additionally, ssGSEA was used to assess the cancer immunity cycle based on the gene expression of each sample (27).

### Estimation of tumor microenvironment

We used multiple methods to infer the tumor microenvironment based on the transcriptional profiles. The cibersort and cibersort-abs (https://cibersort.stanford.edu/) method and LM22 gene signature, including 22 immune cell types, were used to quantify the proportions of immune cells in HNSCC samples. Additionally, quanTiseq, MCPcounter, EPIC, and ssGSEA which were integrated into immunedeconv package and GSVA package (28), were also used to evaluate the immune cell infiltration of tumor samples. All immune-related scores were separated into BASP1^high^ and BASP1^low^ groups.

### Cell culture

The HNSCC cell lines purchased from the American Type Culture Collection (ATCC, Manassas, VA, USA) and the Japanese Collection of Research Bioresources (JCRB, Tokyo, Japan) were maintained in our laboratory. HSC3 and HSC4 cells were cultured in DMEM/F12 medium (DMEM and F12 were 1:1 mixed) and MEM medium (Gibco, Grand Island, NY, USA), respectively, supplemented with 10% fetal bovine serum (FBS, BI, Kibbutz Beit Haemek, Israel). Cells were cultured in 5% CO_2_ at 37°C. The cells proved to be free from mycoplasma or cell cross-contamination.

### Transfection of small interfering RNA

The BASP1-siRNAs were bought from GenePharma (Shanghai, China). The sequences were as follows: NC: 5’-UUCUCCGAACGUGUCACGUTT-3’, 5’-UUCUCCGAACGUGUCACGUTT-3’; si-1: 5’-GAGGCAAGCUCAGCAAGAATT-3’, 5’-UUCUUGCUGAGCUUGCCUCTT-3’; si-2: 5’-GAGAAAGCCAAGGAGAAAGTT-3’, 5’-CUUUCUCCUUGGCUUUCUCTT-3’. For siRNA transfection, HSC3 and HSC4 cells were seeded at 2.5 × 10^5^ cells per well on 6-well plates and transfected with siRNA and Lipofectamine 3000 (Invitrogen, Waltham, MA, USA) reagent when close to 60% concentration, following the recommended instructions. After 24 hours, the transfected cells were harvested for further study.

### Cytotoxicity assays

Cytotoxicity was measured by the Cytotoxicity Detection Kit (LDH) (Roche, Mannheim, Germany) according to the manufacturer’s instructions. Briefly, the transfected cells were seeded in black 96-well plates (Corning, NY, USA) at 2,000 - 3,000 cells per well for 24 hours. Cell supernatant was collected and centrifuged at 400g for 5 min and discarded cell precipitation. 100 μl cell supernatant per well was added to a black 96-well plate. Then, 100 μl LDH Reaction Mixture was added to the supernatant. The mixture was incubated at room temperature away from light for 30 min. The absorbance (490 nm) was measured with a multifunctional microplate reader (BioTek, USA). The BCA quantitative method was performed to normalize the number of cells per well.

### Western blot

Cells were washed with icy phosphate-buffered saline (PBS, Pleasanton, CA, USA) and lysed with lysis buffer (CWBIO, Beijing, China) containing 1% protease inhibitor Cocktail (CWBIO) and 1% phosphatase inhibitors (CWBIO). All protein lysates were centrifuged with 10000g at 4°C for 15 min to remove cell precipitates. Protein concentration was measured using the BCA Protein Assay Kit (Thermo Fisher Scientific, Waltham, MA, USA). Then, loading buffer was added to the proteins and cooked at 99°C for denaturation. Proteins were loaded on a 10% SDS PAGE Gel and transferred onto a PVDF membrane (Millipore, Boston, MA, USA). After being blocked with milk, the membranes were incubated with indicated primary antibody at 4°C overnight. The primary antibodies were listed as follows: BASP1 (Bioss, Beijing, China, 1:1000) and GAPDH (Proteintech, Rosemont, IL, USA, 1:2000). The HRP-linked secondary antibody (CST, Danvers, MA, USA 1:3000) was incubated at room temperature for one hour. The chemiluminescence signals were detected by ChemiDoc Touch Imaging System (Bio-Rad, Berkeley, CA, USA).

### Quantitative RT-PCR

Following the manufacturer’s instructions, total RNA was extracted by the RNeasy kit Mini Kit (Qiagen, Dusseldorf, Germany). Briefly, 2μg RNA was transformed into cDNA by reverse transcription reaction with random primers (Vazyme, Nanjing, China). The qPCR was performed on cDNA using TransStart^®^ SYBR Green qPCR SuperMix (TransGen, Beijing, China) on a StepOnePlus™ Real-Time PCR System (Thermo Fisher Scientific). The primers were listed as follows: BASP1 forward: 5’-GCCCAGGAGACCAAAAGTGA-3’, BASP1 reverse: 5’-CCTTGGGTGTGGAACTAGGC-3’; GAPDH forward: 5’-CTCCTCCTGTTCGACAGTCAGC-3’, GAPDH reverse: 5’-CCCAATACGACCAAATCCGTT-3’. GAPDH was used as the endogenous control. The ^ΔΔ^Ct was used to calculate the relative mRNA expression.

### Lipid peroxidation

Cells were stained with 5μM BODIPY C11 (Invitrogen) at 37°C for 10 min. Then, cells were washed with PBS to remove the dye, digested with trypsin and resuspended in 500μl PBS. After that, cells were passed through a 40μm cell strainer (Corning) and analyzed using the 488 nm laser of a flow cytometer (Beckman, Indianapolis, IN, USA) and analyzed by FlowJo V10 software (BD, Franklin Lakes, NJ, USA).

### Glutathione quantification

The 5×10^4^ cells per well were seeded in a 96-well plate and harvested for measurement of glutathione using the GSH and GSSG Assay Kit (Beyotime, Shanghai, China) according to the manufacturer’s protocol. The Glutathione (GSH) and Oxidized Glutathione (GSSG) concentrations were calculated using a standard curve. The calculation formula is as follows: GSH = Total Glutathione - GSSG × 2.

### Reactive oxygen species detection

Cytosolic ROS (cROS) was detected by the Reactive Oxygen Species Assay Kit (Beyotime). Cells were seeded in a 6-well cell culture plate and incubated with PBS containing 10μM DCFH-DA at 37°C for 30min in darkness when the cell concentration reached 80%. Staining cells were collected and washed with PBS. DCFH-DA was detected at the wavelengths of 488nm using flow cytometer.

### Statistical analysis

All the statistical analysis was executed by R software (version 4.2.0) and GraphPad Prism software (version 8.0.1). If the data followed Gaussian distribution, the parametric test (unpaired student’s test, one-way ANOVA, or Pearson correlation) was conducted. Otherwise, the nonparametric test (Wilcoxon rank test or Spearman correlation) was performed. All data was presented as the mean ± s.d. *P* < 0.05 was considered statistically significant.

## Results

### High BASP1 is related to poor survival of HNSCC patients

**Figure 1:**
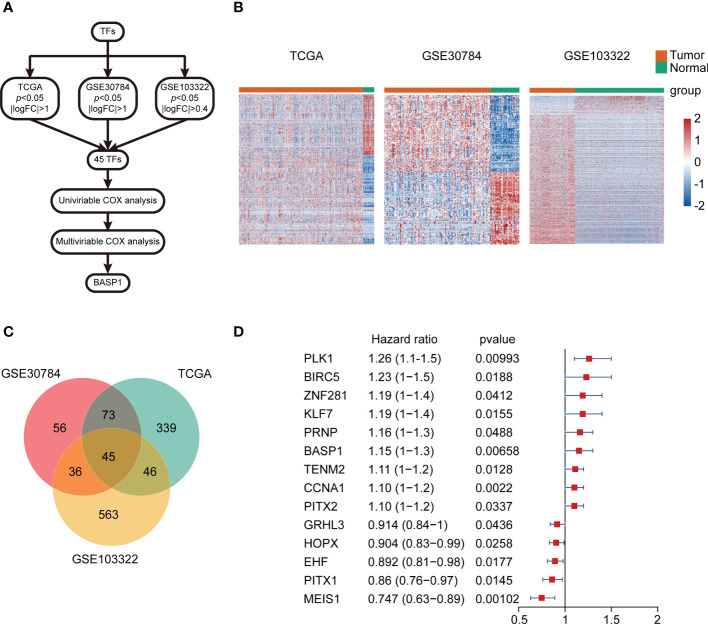
The expressions and prognostic values of TFs in HNSCC. **(A)** Workflow of identifying BASP1 in HNSCC. **(B)** Heatmap of differentially expressed TFs between normal controls and HNSCC tissues in the TCGA database, GSE103322 and GSE30784. **(C)** Venn plot of the intersection of three cohorts of differentially expressed TFs. **(D)** The forest plot of 14 TFs correlated with HNSCC overall survival in the univariate Cox regression model.

The differentially expressed TFs between normal and HNSCC tissues were screened out following the workflow shown in **Figure 1A**. Totally, 45 differentially expressed TFs were identified in HNSCC tissues in TCGA-HNSC, GSE103322 and GSE30784 datasets (**Figures 1B, C**). Univariate Cox regression analysis identified 14 differentially expressed TFs, which were correlated with the overall survival of HNSCC patients (**Figure 1D**). The multivariate Cox regression analysis showed that only BASP1 was associated with patients’ overall survival in either the crude model or the adjusted Model I/II models, indicating that BASP1 was an independent risk factor of HNSCC (**Table 1**).

**Table 1:** Relationships between the expressions of TFs and overall survival of HNSCC patients.

Outcome	Crude Model		Model I		Model II	
	HR (95%)	P-value	HR (95%)	P-value	HR (95%)	P-value
PITX2						
High expression	Reference		Reference		Reference	
Low expression	0.65(0.47-0.9)	0.01	0.67(0.48-0.93)	0.017	0.93(0.29-3)	0.899
CCNA1						
High expression	Reference		Reference		Reference	
Low expression	0.58(0.44-0.76)	<0.0001	0.56(0.43-0.73)	<0.0001	0.57(0.19-1.72)	0.316
BASP1						
High expression	Reference		Reference		Reference	
Low expression	0.64(0.49-0.84)	0.001	0.63(0.48-0.83)	0.001	0.23(0.08-0.64)	0.005
BIRC5						
High expression	Reference		Reference		Reference	
Low expression	0.67(0.5-0.89)	0.005	0.62(0.46-0.83)	0.001	1.46(0.59-3.62)	0.419
PLK1						
High expression	Reference		Reference		Reference	
Low expression	0.64(0.47-0.88)	0.005	0.62(0.45-0.85)	0.003	0.37(0.13-1.01)	0.053
GRHL3						
High expression	Reference		Reference		Reference	
Low expression	1.42(1.07-1.87)	0.014	1.42(1.08-1.87)	0.013	1.1(0.37-3.25)	0.868
EHF						
High expression	Reference		Reference		Reference	
Low expression	1.63(1.2-2.23)	0.002	1.61(1.18-2.19)	0.003	1.39(0.46-4.18)	0.556
TENM2						
High expression	Reference		Reference		Reference	
Low expression	0.67(0.51-0.89)	0.006	0.69(0.52-0.91)	0.009	0.69(0.27-1.75)	0.435
HOPX						
High expression	Reference		Reference		Reference	
Low expression	1.62(1.18-2.22)	0.003	1.7(1.23-2.33)	0.001	0.52(0.15-1.81)	0.307
MEIS1						
High expression	Reference		Reference		Reference	
Low expression	1.62(1.23-2.14)	0.001	1.55(1.17-2.05)	0.002	2.28(0.84-6.16)	0.105
PITX1						
High expression	Reference		Reference		Reference	
Low expression	1.44(1.1-1.89)	0.009	1.43(1.09-1.88)	0.01	1.25(0.4-3.94)	0.702
KLF7						
High expression	Reference		Reference		Reference	
Low expression	0.58(0.41-0.83)	0.003	0.59(0.41-0.84)	0.004	0.39(0.08-1.83)	0.235
ZNF281						
High expression	Reference		Reference		Reference	
Low expression	0.69(0.49-0.97)	0.033	0.66(0.47-0.93)	0.018	1.11(0.35-3.47)	0.859
PRNP						
High expression	Reference		Reference		Reference	
Low expression	0.68(0.52-0.89)	0.005	0.67(0.51-0.88)	0.004	0.55(0.22-1.4)	0.214

Model I adjusted for age and sex.

Model II adjusted for age, sex, alcohol history, HPV, pathologic stage, pathologic t, clinical n.

**Figure 2:**
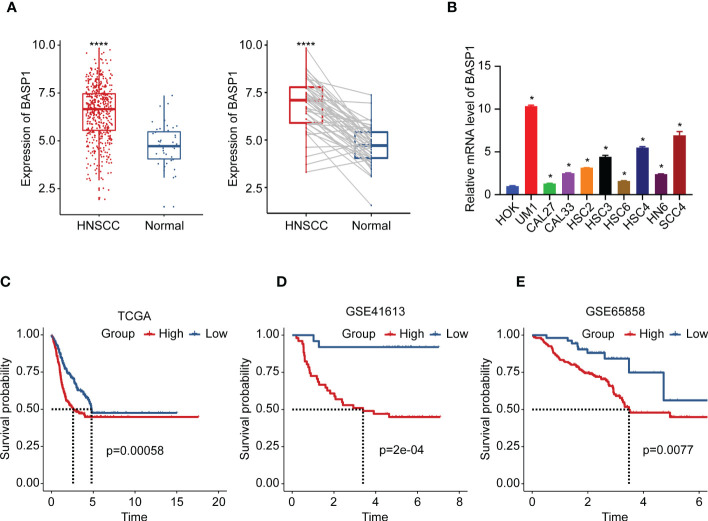
The expression and prognosis of BASP1 in HNSCC. **(A)** The box plot of BASP1 expressions between normal tissues and paired/unpaired HNSCC tissues. **(B)** The mRNA levels of BASP1 in HOK and HNSCC cell lines (UM1, CAL27, CAL33, HSC2, HSC3, HSC6, HSC4, HN6, and SCC4). **(C-E)** Kaplan-Meier survival curve of BASP1 in HNSCC from TCGA HNSC **(C)**, GSE41613 **(D)**, and GSE65858 **(E)**. * p < 0.05; **** p < 0.0001.

In contrast to the normal tissues, the expressions of BASP1 were higher in both unpaired and paired HNSCC specimens (**Figure 2A**). We examined the mRNA levels of BASP1 in human oral keratinocytes cell line (HOK) and HNSCC cell lines using qPCR, and confirmed that BASP1 was upregulated in HNSCC cell lines (**Figure 2B**). The Kaplan-Meier survival analysis illustrated those patients with high BASP1 expression levels were associated with poorer overall survival than those with low expression levels in TCGA, GSE41613 and GSE65858 (**Figures 2C–E**). Meanwhile, BASP1^low^ group had a better prognosis in disease specific survival (DSS) (**Supplementary Figure S1**). Thus, these results demonstrated that BASP1 upregulation was correlated with unfavorable survival in HNSCC patients.

### High BASP1 level exhibits better response to anti-PD-1 immunotherapy

**Figure 3:**
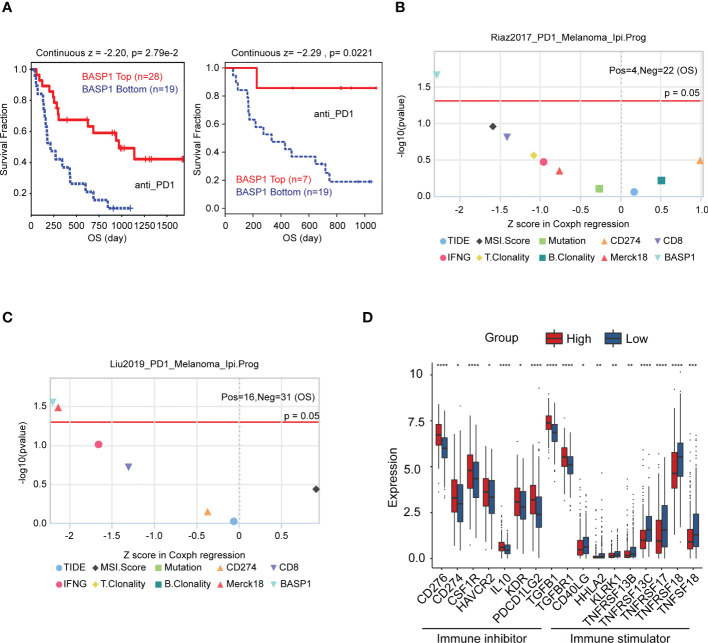
The relationship between BASP1 expression and immunotherapy. **(A)** Overall survival of BASP1^high^ group and BASP1^low^ group in melanoma patients that accepted anti-PD1 therapy. **(B, C)** The evaluation of immunotherapy biomarkers in TIDE. **(D)** Boxplot of the immune molecules expressions in BASP1^high^ and BASP1^low^ HNSCC patients. * p < 0.05; ** p < 0.01; *** p < 0.001; **** p < 0.0001.

Then, we wondered whether BASP1 expression was associated with immunotherapy response. The TIDE algorithm was performed to predict the response rate to immunotherapy, which demonstrated that patients with high BASP1 had a better prognosis in anti-PD-1 therapy in melanoma (**Figure 3A**). The TIDE biomarker evaluation modular showed that BASP1 was potential to stratify immunotherapy patients for precision therapy compared to other indicators (**Figures 3B, C**). Furthermore, the immunoinhibitors (CD276, CD274, CSF1R, HAVCR2, IL10, KDR, PDCD1LG2, TGFB1 and TGFBR1) were high expressed, while the immunostimulators (CD40LG, HHLA2, KLRK1, TNFRSF13B, TNFRSF13C, TNFRSF17, TNFRSF18, and TNFSF18) were low expressed in BASP1^high^ HNSCC patients (**Figure 3D**). Therefore, these results demonstrated that BASP1 was correlated with immune activity, and patients with high BASP1 levels exhibited favorable immunotherapy response rates.

### BASP1 reduced the CD8^+^ T cells infiltration into the tumor microenvironment of HNSCC

**Figure 4:**
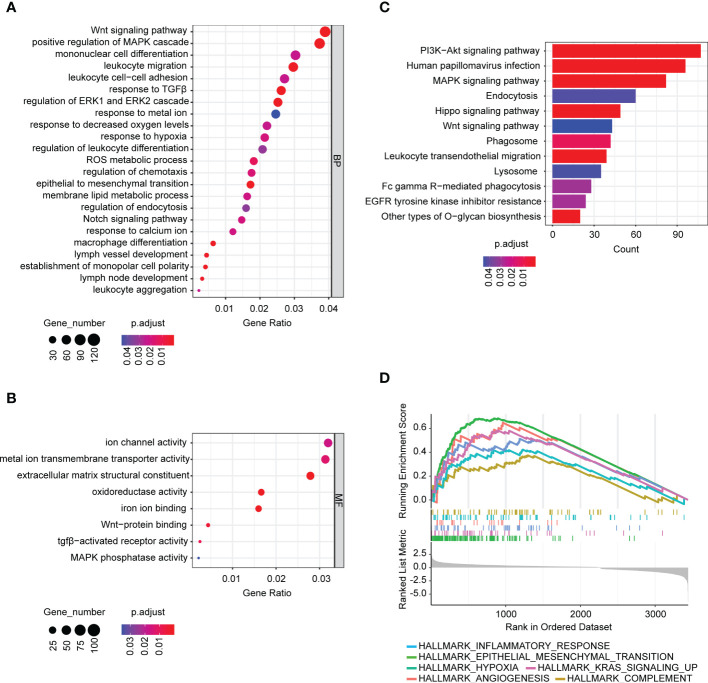
The signaling pathways associated with BASP1 expressions. **(A, B)** Bubble plot of GO analysis of differentially expressed genes between BASP1^high^ and BASP1^low^ group, including biological processes **(A)** and molecular functions **(B)**. **(C, D)** KEGG **(C)** and GSEA **(D)** analysis of differentially expressed genes between BASP1^high^ and BASP1^low^ group.

To elucidate the roles of BASP1 in HNSCC progression and the mechanism of enhancing immunotherapy response, we detected the differentially expressed genes between BASP1^high^ and BASP1^low^ groups. The GO, KEGG and GSEA analyses were performed to examine the different pathways enrolled in the two subtypes. GO and KEGG analysis showed that differentially expressed genes were enriched in several oncogenic signaling pathways, such as Wnt and MAPK signaling pathways, and immune-related signals, such as leukocyte migration, macrophage differentiation, and monopolar cell polarity (**Figures 4A–C**). Besides, GSEA demonstrated that BASP1 was positively correlated with angiogenesis, KRAS, EMT, hypoxia, complement and inflammatory response (**Figure 4D**).

**Figure 5:**
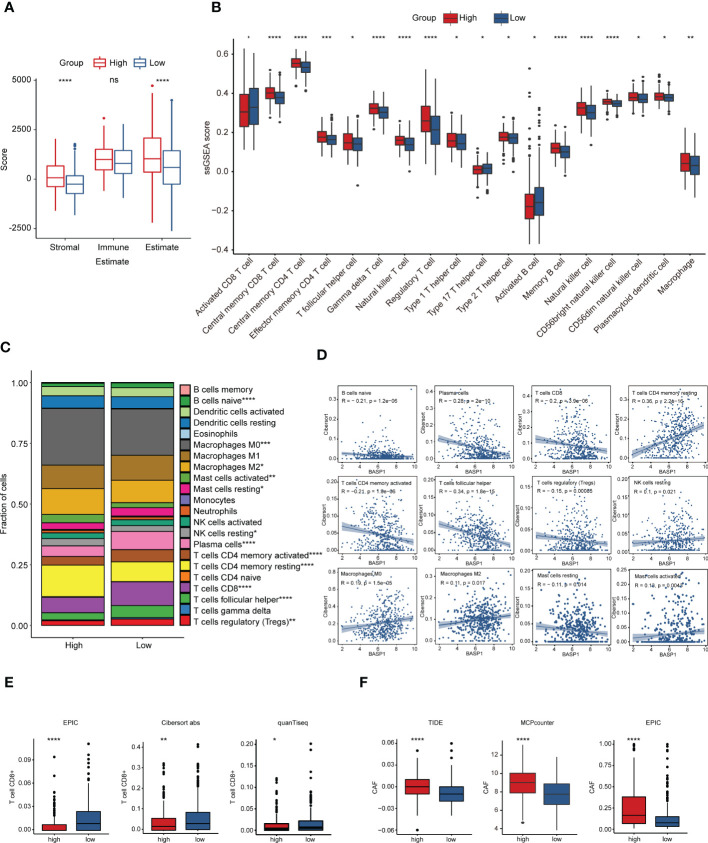
Immune characteristics in different subtypes based on BASP1 expression in HNSCC. **(A)** The immune score and stromal score in BASP1^high^ group and BASP1^low^ group determined using ESTIMATE. **(B)** Boxplot of immune cells infiltration with ssGSEA score in BASP1^high^ group and BASP1^low^ group. **(C)** The fractions of immune cells in BASP1^high^ group and BASP1^low^ group. **(D)** The correlations between BASP1 expressions and cell proportions. **(E)** Boxplot of the CD8^+^ T cells infiltration evaluated by EPIC, CIBERSORT-ABS and quanTiseq. **(F)** The stromal cell fractions in TME with TIDE, MCPcounter and EPIC. * p < 0.05; ** p < 0.01; *** p < 0.001; **** p < 0.0001.

TME plays a fundamental role in tumor progression and immunotherapy response. To explore the correlation between BASP1 expression and TME, we firstly determined the difference in HNSCC TME cell infiltration components between BASP1 subtypes. ESTIMATE analysis showed that the stromal and estimate scores of the BASP1^high^ group were higher than the BASP1^low^ group, yet the immune score was not statistically significant, indicating no difference in total immune cells infiltration between BASP1^high^ and BASP1^low^ patients (**Figure 5A**). Then, we calculated the differences of subgroups of immune cells according to BASP1 expressions using ssGSEA analysis, which clarified that the activated CD8^+^ T cells were reduced in BASP1^high^ group though most immune cells were increased in BASP1^high^ group (**Figure 5B**). CIBERSORT also confirmed that the proportion of CD8^+^ T cells was obviously downregulated in BASP1^high^ group (**Figure 5C**). Furthermore, the expression of BASP1 was negatively correlated with CD8^+^ T cells infiltration (**Figure 5D**). Given that CD8^+^ T cell is the critical mediator of cytotoxic effector in killing tumor cells (29), to further illustrate the killing power of BASP1^high^ tumor, the quenTiseq, EPIC, and Cibersort-ABS were applied to assess the infiltration of activated CD8^+^ T cells. Similarly, all the algorithms clarified that the activated CD8^+^ T cells were lower in the BASP1^high^ group (**Figure 5E**). In addition, cancer-associated fibroblasts (CAFs), which act as stromal cell clusters and promote the recruitment and activation of immunosuppressive cells (30), were substantially elevated in BASP1^high^ tumors (**Figure 5F**). Collectively, these results indicated that BASP1^high^ patients had decreased activated CD8^+^ T cells infiltration and immunosuppressive TME in HNSCC.

### BASP1 inhibited the priming and activation of CD8^+^ T cells

**Figure 6:**
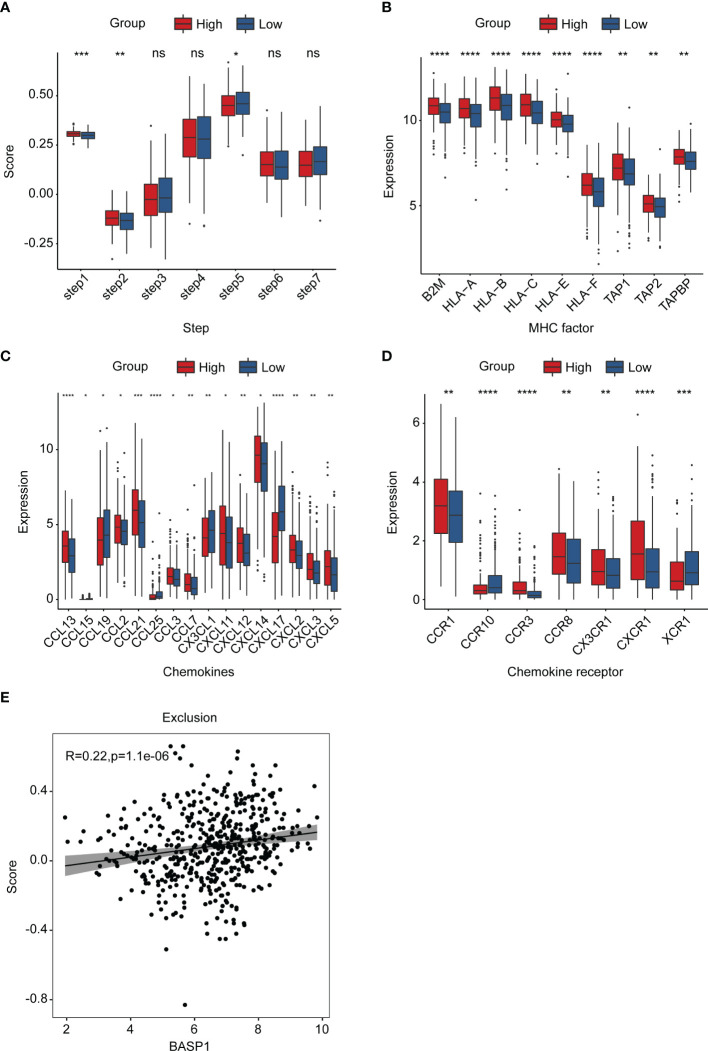
The landscape of BASP1 in the immunity cycle. **(A)** Characteristics of immune microcirculation in BASP1^high^ group and BASP1^low^ group. **(B-D)** Boxplot of the antigen-presenting molecule MHC **(B)**, chemokine **(C)** and chemokine receptor **(D)**. **(E)** The relationship between BASP1 expression and immune escape. * p < 0.05; ** p < 0.01; *** p < 0.001; **** p < 0.0001. ns, means no significance.

Generally speaking, immune cells kill tumor cells in a process with seven steps, including the cancer cell antigens releasement (step 1), cancer antigen presentation (step 2), priming and activation (step 3), trafficking of immune cells to tumors (step 4), infiltration of immune cells into tumors (step 5), recognition of cancer cells by T cells (step 6), and killing of cancer cells (step 7) (31). To explore why activated CD8^+^ T cells were reduced in the BASP1^high^ group, we investigated which step in the immune cycle was disrupted. The ssGSEA was applied to score each step of the immune circulation. As the results showed, although the release of cancer antigens (step1) and cancer antigen presentation (step2) were enhanced in BASP1^high^ patients, there was no significant difference in the priming and activation (step 3) was identified between these two groups, indicating that the reduced CD8^+^ T cells infiltration was due to a dysfunction of immune cell priming and activation activity in BASP1^high^ patients (**Figure 6A**). At the same time, a series of major histocompatibility complex (MHC) molecules and chemokines and their receptors were enhanced in the BASP1^high^ group, which excluded the possible interruption of other steps (**Figures 6B–D**). Moreover, TIDE analysis of the T cell’s functional changes in TME showed a positive correlation between exclusion score and BASP1 expressions, indicating that the cytotoxic function of T cells was abnormal. The ability of tumor cells evading from immune cells was enhanced in BASP1^high^ patients (**Figure 6E**). Thus, these outcomes demonstrated that high expression of BASP1 was correlated with T cell dysfunction and immune escape.

### The immunosuppressive effect of BASP1 is associated with its repression effect on ferroptosis in HNSCC

**Figure 7:**
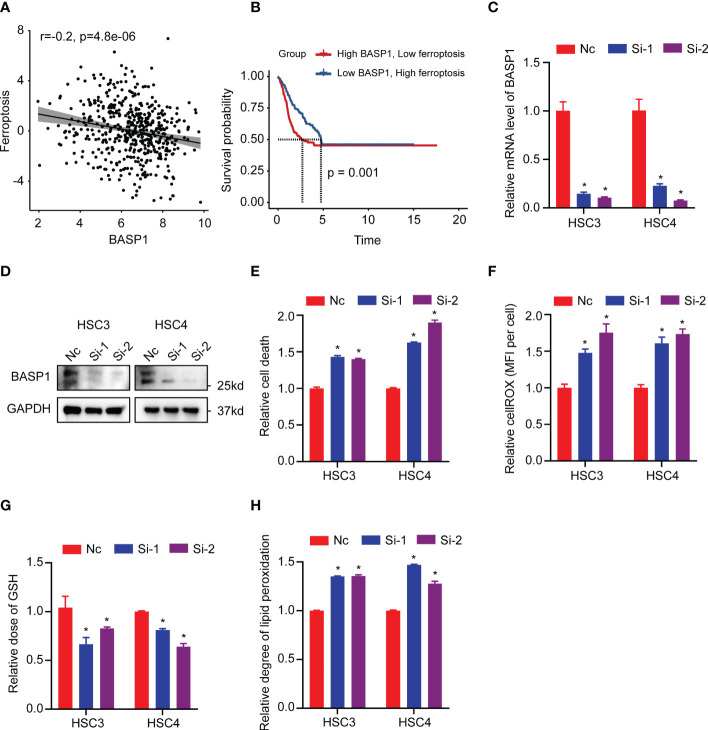
Relationship between BASP1 and ferroptosis. **(A)** The correlation between ferroptosis score and BASP1 expression. **(B)** Survival curves of ferroptosis scores in BASP1^high^ group and BASP1^low^ group. **(C, D)** The mRNA **(C)** and protein **(D)** levels of BASP1 in HSC3 and HSC4 cells transfected with BASP1 siRNAs. **(E–H)** Relative LDH **(E)**, cROS **(F)**, GSH **(G)** and lipid peroxidation **(H)** of HSC3 and HSC4 cells transfected with BASP1 siRNAs. * p < 0.05.

The GO enrichment analysis indicated that the differentially expressed genes between BASP1^high^ and BASP1^low^ tumors were correlated with response to lipid metabolism, hypoxia, metal ion, ROS metabolism and oxidoreductase activity (**Figure 2A**), which are the features of ferroptosis. Considering the critical role of ferroptosis in immune responses (32–34), we wondered whether BASP1 regulated the TME *via* ferroptosis in HNSCC. The ferroptosis score showed a negative correlation with BASP1 expression (**Figure 7A**). HNSCC patients with higher ferroptosis scores had better prognosis (**Figure 7B**). Then, we examined the effect of BASP1 on ferroptosis in HNSCC cells. We transiently knocked down BASP1 in HSC3 and HSC4 cell lines using siRNAs according to the mRNA expression of BASP1 (**Figures 7C, D**). LDH assays indicated that silencing BASP1 increased cell death in HNSCC cells (**Figure 7E**). The ROS contents detected by fluorescent dyes DCFH-DA were obviously elevated in the si-BASP1 groups (**Figure 7F**). Meanwhile, knocking down BASP1 inhibited GSH synthesis (**Figure 7G**). Lipid peroxidation detected by C11-BODIPY staining was substantially increased in the si-BASP1 group compared to the control group (**Figure 7H**). Additionally, we analyzed the relationships between BASP1 and ferroptosis signature genes. We found that BASP1 was positively correlated to ACSF2, ACSL3, FTH1 and CD44, which have been reported to inhibit ferroptosis, and negatively correlated to ALOX12, ALOX15, GLS2, PEBP1 and GOT1, which could enhance ferroptosis (**Supplementary Figure S2**). Hence, these data illustrated that BASP1 inhibited ferroptosis in HNSCC cells.

## Discussion

In the present study, through comprehensively examining the abnormal expressions of TFs and their correlations with HNSCC patients’ clinical outcomes, we identified BASP1 played critical roles in HNSCC. Patients with high BASP1 expression levels were associated with poor prognosis and favorable anti-PD-1 therapy responses. Mechanistically, BASP1^high^ tumors were along with weakened priming and activation of CD8^+^ T cells in TME and low ferroptosis signature. Together, our results demonstrated that BASP1 could suppress tumor cell ferroptosis and induce an alteration of immune TME, which might be a predictive biomarker for antitumor immunotherapy.

BASP1 was initially identified as a plasma membrane-bound, cytoplasmic and cytoskeleton-associated protein from brain extracts, which is recognized to be essential in axon regeneration and neuronal plasticity (35). It is also widely expressed in other tissues and could translocate into the nucleus to interact with other TFs to drive cell differentiation processes (36). Recently, BASP1 was found to be aberrantly expressed in different cancers and implicated in regulating cell proliferation, metastasis, apoptosis and angiogenesis and acted as either a tumor suppressor or oncogene (37–40). For instance, BASP1 hypermethylation and downregulation in hepatocellular carcinoma were considered as a biomarker for early detection (41). In breast cancer, BASP1 could enhance the anticancer effects of tamoxifen treatment, and patients with high BASP1 were associated with better prognosis (42). Besides, BASP1 upregulation was considered as a high-risk factor in lung adenocarcinoma, cervical cancer, as well as in HNSCC (38, 43). Nevertheless, the biological roles and mechanism of BASP1 in regulating HNSCC progression remain unclear. In this study, we identified that BASP1 was upregulated in HNSCC patients. Higher BASP1 level was correlated with poorer survival and better anti-PD-1 immunotherapy response rate.

TME is a vital factor for clinicians choosing immunotherapy strategies. Most HNSCC tumors exhibit highly immunosuppressive3TME, where immunosuppressive factors promote immunosuppressive cell recruitment and inhibit the antitumor effects of immune-activated cells (8). Increased infiltration of CD8^+^ T cells and NK cells has been recognized to be associated with improved survival, whereas elevated infiltration of Treg cells, M2 macrophages and neutrophils are related to advanced disease and poor clinical outcomes (44). Upregulation of immune checkpoint inhibitors (e.g., PD-1/PD-L1) attenuates the cytolytic activity of CD8^+^ T cells in HNSCC (45). Here, we found that, although there was no significant difference in the total immune score between BASP1^high^ and BASP1^low^ tumors, BASP1^high^ ones exhibited reduced and exhausted cytotoxic CD8^+^ T cells, which have critical influence on immune checkpoint blockade therapy efficacy (46). Furthermore, the increased immune checkpoint inhibitors and reduced immune checkpoint stimulators in the BASP1^high^ group also indicated an exhausted environment (47). Moreover, step3 in the cancer-immunity cycle revealed a blockage of T cell activation in BASP1^high^ tumors. Thus, high BASP1 expression might negatively influence the activity of immune cells.

The immunosuppressive TME of HNSCC is governed by multiple immune regulatory pathways, which provide the rationale for combinatorial strategies in patients with HNSCC (45). Previous studies mentioned that tumor cells could trigger antitumor immunity by inducing immunogenic cell death, including ferroptosis, necroptosis and pyroptosis. ICIs coordinated with immunogenic cell death could achieve better outcomes even in ICI-resistant tumors (48). We clarified that BASP1^high^ tumors exhibited suppressed ferroptosis signature. Silencing BASP1 could substantially induce ferroptotic tumor cells in HNSCC. Ferroptotic cancer cells can release several immune-stimulating signals, allowing immune cells to infiltrate tumors (49). Furthermore, the increased immunogenicity of ferroptotic cancer cells has been reported to induce tumor-specific immune responses, enhancing the efficacy of anti-PD-1/PD-L1 therapy (50). Thus, we proposed that it could be possible to utilize BASP1 as a therapeutic target or biomarker for stratification in HNSCC immunotherapy, which needs further elucidation.

However, there are several limitations existed in this study. For example, the prediction effect of BASP1 on the response rate to immunotherapy was performed in melanoma, which need to be further studied in HNSCC patients in the future. The mechanism of BASP1 suppressing ferroptosis to influence TME in vitro and in vivo need to be further validated.

## Conclusion

In conclusion, we identified BASP1 as a poor prognostic biomarker for HNSCC, revealed its potentiality in predicting immunotherapy response, and offered a novel candidate for stratifying patients in immunotherapy of HNSCC. We pointed out that combining BASP1 inhibition induced ferroptosis might be a potential therapeutic method for overcoming immunotherapy resistance.

## Data availability statement

The datasets presented in this study can be found in online repositories. The names of the repository/repositories and accession number(s) can be found in the article/**Supplementary Material**.

## Author contributions

XR, JX and BC were responsible for conception and design and supervised the project. XP and XX performed the bioinformatics analysis. XP and LW conducted the experiments. SZ, RY, YC, XC participated in collecting data and helped to draft the manuscript. All authors contributed to the article and approved the submitted version.
