# Supplementary material for: BASP1 is a prognostic biomarker associated with immunotherapeutic response in head and neck squamous cell carcinoma
Source: Front Oncol. 2023 Jan 27;13:1021262. doi: 10.3389/fonc.2023.1021262 (PMC9911441; doi:10.3389/fonc.2023.1021262)
Supplement: Supplementary file 1 [file DataSheet_1.docx]

Supplementary materials


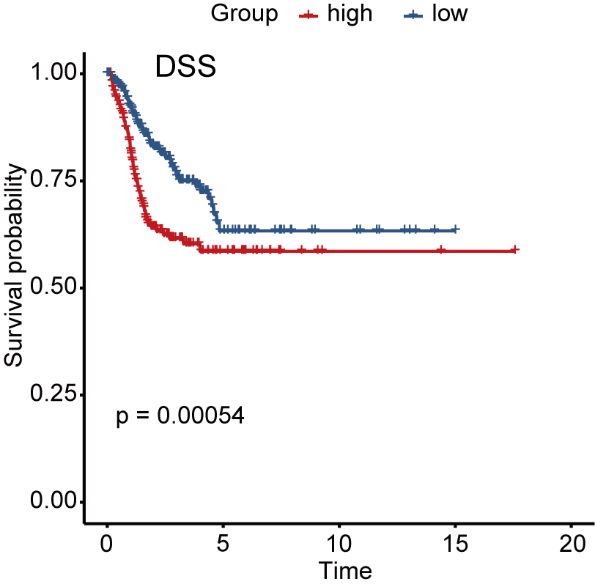


Supplementary Figure S1. Analysis of the expression of BASP1 and disease specific survival (DSS).


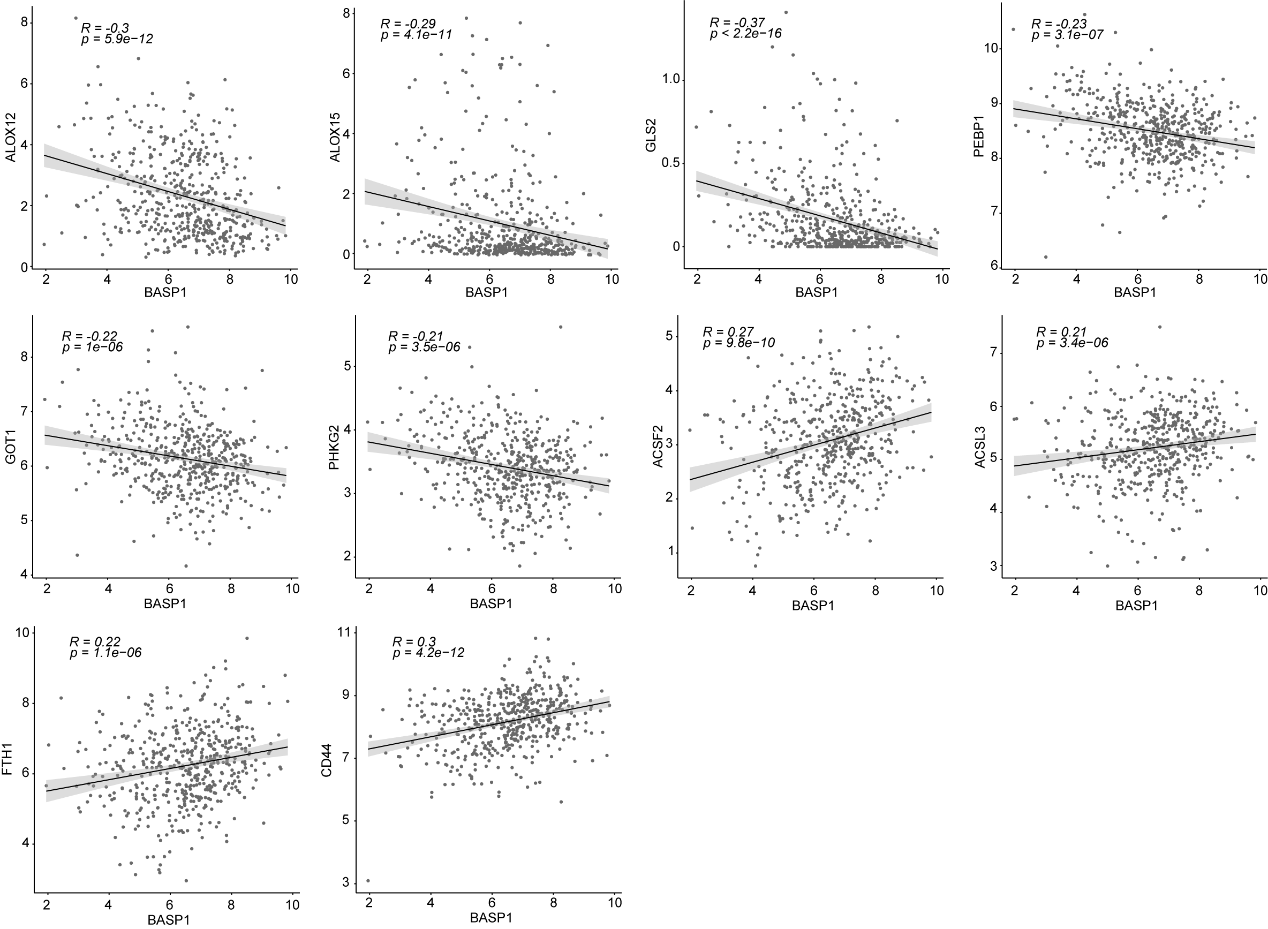


Supplementary Figure S2. The relationship between BASP1 and ferroptosis signatures. The expression of BASP1 positively correlated to the expression of ferroptosis related signatures, including ACSF2, ACSL3, FTH1 and CD44, while negatively correlated to ALOX12, ALOX15, GLS2, PEBP1 and GOT1.
